# 

**Published:** 1898-02-05

**Authors:** 


					TRE Hospital. "The standard of highest purity."?Lancet. Feb. 5th, 1898.
CADBURY'S ?F CADBURY'S
COCOA Irl /It COCOA
NO DRUGS OR ALKALIES USED,
S. Vol. xxm. [jssisia Saturday,
Feb. 5th, 1898.
? -^NORFOLKTuaWtn tY/CH HOSPLTAL
j-SuhscnptionTenns,] pRICE TWOPENCE.

				

